# Health System Resiliency and the COVID-19 Pandemic: A Case Study of a New Nationwide Contingency Staffing Program

**DOI:** 10.3390/healthcare10020244

**Published:** 2022-01-27

**Authors:** Shay Cannedy, Alicia Bergman, Melissa Medich, Danielle E. Rose, Susan E. Stockdale

**Affiliations:** 1 VA HSR&D Center for the Study of Healthcare Innovation, Implementation & Policy, VA Greater Los Angeles Healthcare System, Los Angeles, CA 90073, USA; alicia.bergman@va.gov (A.B.); melissa.medich@va.gov (M.M.); danielle.rose@va.gov (D.E.R.); susan.stockdale@va.gov (S.E.S.); 2 Department of Psychiatry and Biobehavioral Sciences, UCLA, Los Angeles, CA 90095, USA

**Keywords:** telehealth, COVID-19, resilience, health systems, workforce wellbeing

## Abstract

When COVID-19 emerged, the U.S. Veterans Health Administration (VA) was in the process of implementing a national contingency staffing program called Clinical Resource Hubs (CRHs). CRHs were intended to provide regional contingency staffing for primary and mental health clinics experiencing staffing shortages primarily through telehealth. Long-term plans (year 2) included emergency management support. Early in the implementation, we conducted semi-structured interviews with CRH directors and national program leaders (n = 26) and used a rapid analysis approach to identify actions taken by CRHs to support the resiliency of the VA healthcare system during the pandemic. We found that the CRH program was flexible and nimble enough to allow VA to leverage providers at hubs to better respond to the demands of COVID-19. Actions taken at hubs to sustain patient access and staff resiliency during the pandemic included supporting call centers and training VA providers on virtual care delivery. Factors that facilitated CRH’s emergency response included hub staff expertise in telehealth and the increased acceptability of virtual care among key stakeholders. We conclude that hub providers serving as contingency staff, as well as specialization in delivering virtual outpatient and inpatient care, enabled VA health system resiliency and recovery during the COVID-19 pandemic.

## 1. Introduction

Caring for patients with COVID-19 has tested the resiliency of health care systems in the United States and globally [1,2,3]. In January 2020, the World Health Organization (WHO) declared coronavirus disease 2019 (COVID-19) an international public health emergency. By March 2020, the United States had become the hardest hit country in the world, with 82,404 confirmed cases that severely strained health care systems in hot spot areas [4]. A key question within health policy and systems literature is how health systems can become more resilient in the face of extraordinary circumstances like an international pandemic. Within a service industry such as healthcare, labor resiliency is particularly critical. As the U.S.’s largest national integrated healthcare system, the Veterans Health Administration provides an opportunity to examine the relationship between providing critical support through a contingency staffing program for primary care and mental health teams and achievement of organizational resiliency needed to respond effectively to a national pandemic.

We define health system resilience as the capacity of health actors and institutions to effectively respond to and recover from crises (e.g., pandemics, natural disasters, political insurgencies) while maintaining core functions [5]; (cf. [6,7]). Kruk et al. [5] identify five characteristics of resilient health systems: they are aware of system strengths and vulnerabilities, they address diverse health needs, they self-regulate by containing health threats while continuing care delivery, they integrate diverse actors and ideas to solve health crises, and they are adaptive and able to improve performance in crisis and non-crisis times. Ultimately, a health care system is considered resilient if life is preserved and positive medical outcomes are achieved for all during and after a crisis [5].

Earlier literature establishes the critical role of sufficient staffing in healthcare, especially during times of crises. Having sufficient clinical staffing is positively associated with the quality of preventative care [8,9] and lower staff burnout [10,11]. During crises, having an adequate and capable workforce is also crucial for health system resilience due to healthcare workers’ role as first responders [7,12,13]. Specifically, prior research indicates the need for a flexible workforce with a diverse skill set that can collaborate across various sectors (state, private, and non-profit) [14,15].

While health systems researchers have advanced conceptual thinking about the meaning of “resilience”, specific suggestions regarding how systems can become more resilient remain scarce. This study seeks to build on the evidence of improving healthcare systems resilience during crises, given the number of people living in fragile contexts where natural or human-made crises are common [13]. Moreover, additional work is needed on strategies for developing a resilience-oriented workforce that is responsive to public healthcare needs during a crisis. This study seeks to fill this evidence gap through the consideration of a new, nationwide program launched in October 2019 at the VA Healthcare System called Clinical Resource Hubs (CRHs). CRHs provide contingency staffing for primary care and mental health services by using predominantly telehealth modalities in 18 regional networks. We describe how CRH resources (e.g., additional primary care providers with telehealth expertise) were leveraged during the early days of COVID-19 to deliver patient care, workforce training and support during the pandemic, and the key factors that facilitated these responses. We hypothesize that having flexible contingency providers and staff who specialize in telehealth modalities may increase a system’s resilience, while also providing the needed contingency care during non-crisis times.

## 2. Methods

### 2.1. Clinical Resource Hub Program

VA is characterized by its diverse operating environments, large complex patient population, and its role as a back-up system to the private sector in times of national emergency [16]. Comprised of more than 1000 clinics located within 139 healthcare systems in 18 regional networks, VA has a substantial presence across large metropolises, suburbs, and rural areas, offering a wide spectrum of care (e.g., primary care, nursing home care, substance abuse, specialized procedures).

Like other healthcare systems, VA has wrestled with chronic shortages in primary care and mental health providers, especially in rural areas [17]. To mitigate the impact of these shortages on patient access to care, VA implemented a regional healthcare delivery model as a national program in 2019 called Clinical Resource Hubs. CRHs emerged out of previous telehealth pilot programs that had demonstrated improved patient outcomes [18].

**Figure 1:**
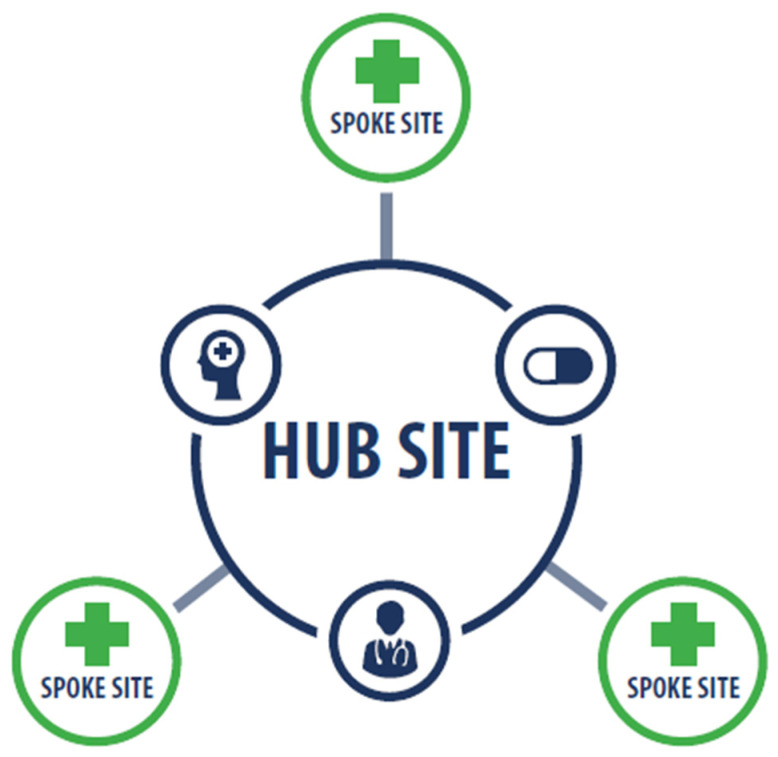
CRH Hub and Spoke Model.

CRHs offer clinical staffing resources using a combination of telehealth and in-person care delivery. Through a hub and spoke model (Figure 1), CRH-hired staff primarily provide clinical contingency coverage for primary care and mental health services at VA facilities (medical centers and community-based outpatient clinics) in their regional network. In the first year of program implementation, a total of 636 clinicians were hired across 18 stand-alone hubs. Clinical contingency staffing coverage at spoke sites is temporary (in most cases no longer than two years), during which time sites are expected to recruit permanent staff. Most spoke sites are community-based outpatient clinics, although hubs also provide care to patients at VA medical centers.

The VA Office of Primary Care and a CRH Advisory Council developed an implementation framework that included 10 key elements outlining the structure and functioning of the CRHs, as has been described elsewhere. In addition to providing contingency staffing predominantly through telehealth, the implementation framework specified that CRHs would be prepared to support emergency management services by 2022. A key factor in VA’s capability to deliver telehealth services for this program is the 2018 Anywhere to Anywhere legislation which overrides state restrictions on licensing and state-specific telehealth laws and allows VA providers to deliver care to patients in clinics across state lines and to patients in the home [16].

### 2.2. Sample and Data Collection

Data were collected as part of a larger six-year formative evaluation of the CRHs initiated in 2019 by the VA Office of Primary Care and the CRH Advisory Council. The first year of the evaluation included assessing implementation based on qualitative CRH program data (e.g., interviews with national and regional leaders), an organizational survey, and VA administrative data.

Findings presented here draw on semi-structured telephone interviews with 26 key stakeholders. Stakeholders include 18 regional hub directors representing 17 of the 18 VA regional networks, and eight national program leaders. As a formative evaluation of a new program, interview questions focused on early-stage implementation progress (including barriers and facilitators), key partnerships, processes for setting up clinics to receive services, future emergency planning and response, and competing priorities for regional leadership. For this paper, responses to the following questions were analyzed: Has the CRH been involved with your region’s COVID-19 response? Has the COVID-19 response changed the way you are doing things? How so? What barriers or facilitators to implementation (if any) did the pandemic create? And have you created any other new partnerships to address the pandemic?

Interviews, ranging from 45–60 min, were conducted between June 2020 and April 2021, starting nine months after national program implementation. A team of trained interviewers provided appropriate confidentiality assurances and verbal consent to audio-record was obtained from participants before the start of each interview. All interviews were recorded and transcribed.

### 2.3. Data Analysis

Using a rapid analysis approach [19,20], four members of the project team completed summaries of interviews using a template based on interview guide domains. Each summary was cross-validated by the interviewer following the interview and discrepancies were resolved through discussion and consensus, or flagged for later validation against interview transcripts. After summaries were completed and validated for each interview, three team members randomly audited half (50%) of the summaries, where they were checked against transcripts for accuracy and completeness (e.g., included main issues in the data). Next, team members synthesized across the summaries by role (e.g., hub director interviews). During this process, analysts took deductive and inductive approaches. A deductive template based on the interview guide domains was used to structure analysis, but analysts also highlighted other issues that emerged inductively from the data. The analysis was completed with consensus checks among team members to identify major themes from key interview domains (e.g., local CRH implementation status, barriers to implementation). After the formation of initial themes, themes were compared against each other and the original summaries they were generated from. The themes were refined, and those with similar concepts were grouped together to form overarching themes. Finally, working from the hub director and national program leader synthesis documents, the lead author identified and compiled themes and sub-themes related to CRH responses to COVID-19, as well as associated quotes from transcripts. Resulting themes and quotes were reviewed and validated by two members of the project team.

## 3. Results

Respondents reported that CRHs assisted with VA’s COVID-19 response by maintaining patient access to care during the pandemic. This was accomplished by providing contingency staffing for outpatient and inpatient care and triage support at VA spoke sites and non-spoke sites, as well as in the private sector. In addition, respondents reported that CRHs provided workforce support through training, coaching, and care delivery. Finally, respondents discussed key factors that facilitated CRHs’ ability to quickly respond to the pandemic.

### 3.1. CRHs Maintained Patient Access to Care during the Pandemic

#### 3.1.1. Outpatient Clinical Coverage

Six CRH directors and program leaders reported that CRHs provided virtual contingency providers for patient care in the areas of primary care, mental health, and specialty services when regular providers were out due to COVID-19 related issues. This included virtual outpatient staffing coverage for primary care providers and nurses reassigned to hospitals during the surge; coverage for primary care teams dealing with provider attrition hastened by COVID-19; and, in one case, coverage for a team who lost a provider to COVID-19. For example, hub directors reported:

*So when COVID-19 started we actually proactively connected with our spoke sites and told them, ‘We committed to this 100% telehealth provider for you so, we recommend that you shift your in-person providers to assist the main facility’, because a lot of facilities were shifting their outpatient providers to inpatient care depending on where they were as a hot spot or not. And then we also suggested that if they had Veteran needs, from other [small clinics], then to map those Veterans to our CRH provider panel.*

(CRH director A)

*We actually put two primary care providers in there to take over a team because they lost a provider out to COVID-19. And so we’ve got those folks in there actually just doing phone calls and video visits covering for them. So you know, [we say], ‘We’re here to help. I know you’re having access issues. Let us jump in.’*

(CRH director B)

#### 3.1.2. Inpatient Coverage

In addition to outpatient coverage, two CRH directors described how some CRH providers delivered virtual coverage for inpatient units in social work and pharmacy, where they helped with end of life care (counseling and orders) and medication orders:

*We detailed […] full time social workers virtually […] to cover inpatient care at our VA, which was considered one of the top hot spots in the country for COVID-19. So they actually worked 100% remotely from their home or their existing facilities to cover all inpatient units. And they saw those Veterans either by phone or video on demand, which was nice because they could also include their family member in the visit. It went really well.*

(CRH director A)

*…we have a pharmacist that was detailed to [City], virtually, to assist with providing taking care of inpatient drug orders on patients when they had people that were out.*

(CRH director B)

#### 3.1.3. Triage Support

Respondents (n = 12) described how CRHs also assisted with the pandemic response by providing virtual triage and episodic care for clinics and/or nurse advice lines. For instance, one director stated:

*The* *nurses actually started a kind of mini-COVID clinic where if people were identified as COVID positive and their sites didn’t have enough bandwidth to call and check on them for the five days after they had a positive reading, our team did that.*

(CRH director C)

CRH providers also assisted nurse advice lines by fielding COVID-19 and non-COVID-19 calls in the areas of primary care and mental health, which helped divert visits from the emergency room. A director explained:

*We actually detailed a few LIPs [licensed independent practitioners], I believe like 10 to 15, to work on second-level triage support to our clinical contact center [nurse advice line] during off hours, so the evening hours and weekends. They took second-level triage calls for COVID and flu-related symptoms.*

(CRH director A)

In another region, the CRH set up a virtual “next day program” to see non-COVID-19 patients referred by the nurse advice line.

#### 3.1.4. The Provision of Patient Care in the Private Sector

Our qualitative findings show that CRH was part of the pandemic response that extended beyond VA as part of VA’s mandate to provide back-up care to the wider community during national emergencies, referred to as VA’s “fourth mission”. Three CRH directors reported providers answering the White House COVID-19 phone line, vaccinating airport personnel, and providing virtual outpatient mental health care in non-VA primary care clinics.

Thus, CRH providers served as emergency contingency providers although they were deployed to settings that were not part of the original plan of serving spoke sites. Moreover, it was advantageous that providers were versatile enough to easily pivot to virtual inpatient and outpatient care.

### 3.2. CRH Providers Provided Workforce Care and Training during the Pandemic

Given their virtual and clinical care expertise, four directors stated that CRH providers also aided VA’s pandemic response by providing training and care to the VA workforce. Specifically, they trained non-CRH providers how to deliver virtual care, thereby increasing capacity for treating patients when clinics were closed to in-office visits. A CRH director explained:

*Since we were [using] video [in our CRH work], we were able to help our region by just showing them how it worked for us and that it’s not a scary technology but one that’s, you know, could be beneficial. […] You know, we shared just best practices such as the need to obtain consent and locations and all that fun stuff.*

(CRH director D)

CRH mental health providers also provided support to their non-CRH colleagues for COVID-19-related work and health issues. Two CRH directors reported:

*We’ve also offered services in terms of providing coaching and counseling with our providers in the region who may be involved in any palliative care decisions in COVID-19 patients.*

(CRH director E)

*I have a psychiatrist who’s doing second-level screening of patients and employees after hours. So basically he works from 5:30 PM to 5:30 AM taking calls on anybody that screens positive.*

(CRH director B)

The training and care of the VA workforce was not part of the original CRH mission or an anticipated need. However, CRH hub directors were able to deploy CRH providers and staff where needed to build health system resilience by supporting providers in pivoting from face to face to virtual care, supporting palliative care decisions, and following up with COVID-19-positive employees.

### 3.3. Factors Facilitating the CRH Response to COVID-19: Expertise and Acceptability of Telehealth

Respondents described several factors that facilitated CRHs’ capacity to implement their emergency management functions. First, directors and leaders (n = 3) explained that CRH providers’ extant expertise in telehealth (e.g., technology experience, best practices knowledge) coupled with their clinical knowledge enabled them to easily and quickly assist spoke sites and other facilities within and outside of VA during a time of restricted in-person care. For example, a national program leader explained that VA providers required time to switch from in-person to telehealth care delivery when COVID hit, which was not the case for CRH providers.

[CRH providers] *were already ready to do this work […] we were already doing it […] so it did allow for some opportunities to deploy services to places that were really struggling*

(National program leader 8)

Second, respondents stated (n = 4) that positive attitudes toward telehealth among VA stakeholders garnered buy-in and support for CRHs’ emergency management function. Prior to COVID-19, regional and national leaders were skeptical of this modality, but came to accept it as either necessary in the context of stay-at-home orders, or because it was equivalent to face-to-face care. As one national program leader remarked,

*We had some initial resistance in some [regions] to adopt virtual care. But with COVID it forced that function and awakened several leaders to say, ‘Gosh this does work and we can do it and in fact it’s critical for us’*

(National program leader 5)

Thus, the nature of the crisis as a highly transmissible disease requiring social distancing combined with CRH expertise in telehealth facilitated the capacity of CRH to support VA’s pandemic response.

## 4. Discussion

We examine how VA’s CRH program, as a contingency staffing program, was leveraged to assist with the pandemic response within and beyond VA, and factors that facilitated this response. We illustrate how this program, as a regional pool of providers with expertise in telehealth, enabled VA to continue to deliver locally relevant patient care and address workforce needs during a time of stress for healthcare systems. As such, we argue that CRHs aided in system resilience. In particular, we found Kruk et al.’s [5] resilience concepts of adaptiveness (e.g., ability to change to improve function), self-regulation (e.g., ability to contain a threat while delivering core services), and integration (e.g., information sharing and coordination among multiple actors) useful for describing the role of the CRH in VA’s COVID-19 response. In addition, our findings suggest staff wellbeing as another component of health system resilience.

We contend that during the shock of the COVID-19 pandemic, CRH’s flexible human resources enabled VA’s adaptability, allowing for improved responses to a pandemic. Specifically, CRH resources enabled VA to adapt to sudden demand by providing a pool of contingent providers with institutional knowledge and experience, who were redirected to triage support and inpatient coverage. Thus, they are a flexible workforce that can nimbly pivot from routine work covering staff shortages in primary care and mental health clinics to emergency management, rather than costly “extra” human resources that are utilized only during emergencies. Indeed, disaster management literature indicates that effective disaster response is contingent upon the degree of embedded “surge capacity” within a system [13,21]. During the COVID-19 surge, for example, European health care systems sought to increase workforce capacity through various ad hoc measures such as cancelling staff vacations, shifting employees from part-time to full-time, and shifting nursing students to clinical practice [22]. The CRH program represents a different approach: one that augments existing staffing levels within administrative regions through embedded virtual providers who have institutional knowledge and sensitivity to local patient populations.

In addition to adaptability, health system resiliency requires the ability to contain a threat while delivering core services, or self-regulation [5]. At VA, there was early recognition of the need to quickly shift to virtual modes of care to prevent disease exposure, facilitated by the 2018 Anywhere to Anywhere policy that gave VA providers the authority to deliver telehealth across state lines [16]. This flexibility coupled with CRH providers’ extant expertise in telehealth enabled CRH to institute novel service delivery modes for triage and routine care. Indeed, studies indicate that telehealth has an important role to play in emergency responses by facilitating triage, supplying clinical services when facilities are otherwise unable to meet this need, and decreasing the spread of communicable disease [23,24,25]. For example, findings from a retrospective chart review revealed that 15% of patients transported to the hospital by ambulance could be managed through a telemedicine consultation with an emergency physician [25]. However, more evidence is needed to support the widespread use of telehealth generally and to better understand how specific disaster contexts may hinder its utility, such as connectivity issues that disrupted teleurgent care in Japan during the 2011 earthquake [25]. Moreover, telehealth utilization rates on a global scale remain low due to legal barriers, technological issues, and provider and patient lack of familiarity [26].

We also found that CRH helped foster health system integration, another characteristic of a resilient health system [5]. Key components of integration are information sharing and coordination among multiple actors to address public health crises [5]. Our findings indicate that CRH was a conduit for information sharing by providing telehealth training to a diverse group of VA clinicians and support staff, thereby increasing care capacity in a context of social distancing. In addition, CRH helped VA link to the private sector as part of a wider public health response through, for example, vaccinating airport personnel and providing virtual outpatient mental health care in non-VA primary care clinics.

Our findings corroborate elements of Kruk et. al.’s framework [5], but also call attention to staff wellbeing as an additional and important component of resilient health systems, as noted in other literature [27]. For example, in a study of how infrastructure organizations (e.g., water services, roadways, telecommunications) in New Zealand responded to an earthquake, researchers found that resilience was enhanced in organizations that prioritized staff wellbeing. Actions included providing resources needed to do one’s job and actively listening and responding to stress [ibid]. In the context of infectious disease more specifically, there is evidence that counseling provided by employers can have a protective effect on work-related stress [28]. In line with this prior research, we suggest that CRHs may be a useful model for not only delivering patient care during pandemics, but also facilitating employee wellbeing through virtual health screenings and counseling.

## 5. Limitations and Areas for Future Research

This study has several limitations. First, it captured CRH responses early in the pandemic and thus may not reflect the evolving nature of hub responses as conditions changed. In addition, the findings could be biased to emphasize positive impacts of the program and may not be exhaustive of all perspectives related to CRH COVID-19-related work, as our sample was limited to hub directors and national program leaders. And finally, as a study focusing on program implementation, we do not capture patient and workforce outcomes. Future research should focus on gaining frontline perspectives for a more complete picture of the impacts of the CRH COVID-19 response. In addition, it should explore the intervention’s effectiveness while also identifying the most salient resilience factors for future iterations which may guide other organizations looking to protect patients and workforces during the pandemic.

## 6. Conclusions

The CRH program described here was implemented at VA just before the start of the COVID-19 pandemic. As a predominantly virtual, regional solution to staffing shortages, CRHs were also nationally mandated to eventually roll out emergency management services to VA and the private sector in the later years of program implementation. However, the details of its emergency function were fleshed out ahead of schedule when COVID-19 emerged. National program leaders and hub directors recognized the emergency need and were able to reach out to struggling sites and deploy providers where they were greatly needed.

We conclude that having contingent staffing adept at virtual modes of care may enable health system resiliency during public health crises. As a multi-function program, CRHs may be a model for other similarly resourced healthcare organizations for supporting day-to-day operations through virtual staffing solutions, which can be redirected to meet human resource needs during shocks. Thus, CRHs present a hub and spoke model that can not only improve care access in underserved areas, but can also be used as contingency providers during crises to foster healthcare system resiliency.

## Data Availability

The relevant data are contained in the manuscript, and further queries can be addressed to the corresponding author.
